# Effect of physical activity on mental toughness and quality of life in gender-specific participants: a cross-sectional examination

**DOI:** 10.3389/fpsyg.2025.1586278

**Published:** 2025-04-28

**Authors:** Mohammad Ahsan, Abdullah Alzahrani, Abdullah Alhusayni, Maysa Aljohany

**Affiliations:** ^1^Department of Physical Therapy, College of Applied Medical Sciences, Imam Abdulrahman Bin Faisal University, Dammam, Saudi Arabia; ^2^Department of Rehabilitation Sciences, College of Applied Medical Sciences, Shaqra University, Shaqra, Saudi Arabia; ^3^Department of Health Rehabilitation Sciences, Faculty of Applied Medical Sciences, University of Tabuk, Tabuk, Saudi Arabia

**Keywords:** physical activity, mental health, mental toughness, quality of life, sex differences

## Abstract

**Background and aims:**

Mental toughness is a psychological construct related to resilience and perseverance, and quality of life encompasses physical, emotional, and social wellbeing, which are critical indicators of overall health and wellbeing. This study aims to investigate the effects of different levels of physical activity on mental toughness and quality of life in both male and female participants.

**Methods:**

This cross-sectional study consists of 220 participants with the age range of 18–31 years. The data were collected using the International Physical Activity Questionnaire, the Mental Toughness Questionnaire, and the World Health Organization Quality of Life. Gender specific differences were determined using an independent *t*-test (two-tailed). The effect of varying levels of physical activity in male and female participants was determined by analysis of variance.

**Results:**

Significant differences were found between male and female participants for physical activity levels as high (*t* = 4.21, *p* ≤ 0.001, *d* = 871.80), moderate (*t* = 3.72, *p* ≤ 0.001, *d* = 1309.59), and low (*t* = 3.21, *p* = 0.002, *d* = 520.91) physical activity participants. Statistically significant differences were also found between gender for mental toughness parameters [handling pressure (*t* = 17.96, *p* ≤ 0.001, *d* = 1.25), concentration (*t* = 16.07, *p* ≤ 0.001, *d* = 1.22), mental rebounding (*t* = 13.34, *p* ≤ 0.001, *d* = 1.45), winning attitude (*t* = 12.01, *p* ≤ 0.001, *d* = 1.17)] and quality of life parameters [physical (*t* = 13.16, *p* ≤ 0.001, *d* = 1.58), mental (*t* = 6.17, *p* ≤ 0.001, *d* = 1.69), social (*t* = 4.58, p ≤ 0.001, *d* = 2.05), environmental (*t* = 8.35, *p* ≤ 0.001, *d* = 4.95)].

**Conclusion:**

Statistically significant differences were found between genders in levels of physical activity, mental toughness, and quality of life. Future research should aim to unravel the complex interlink between physical activity, mental toughness, and quality of life, using longitudinal designs and diverse populations to validate the findings and explore the underlying mechanisms further.

## Introduction

Physical activity is generally described as any type of body movement that involves skeletal muscles and results in energy consumption due to skeletal muscle action, such as housework and participation in sports and fitness programs (Pucci et al., 2012). Regular physical activity is one of the most effective methods for preventing cardiometabolic risks such as high blood pressure, diabetes, stroke, obesity, osteoarthritis, and sleep apnea (Reiner et al., 2013). It is now globally recognized as a key component of a healthy lifestyle (Muaidi and Ahsan, 2020). Physical activity offers numerous physical, psychological, social, and environmental benefits. In addition to its well-established effects on physical health, growing evidence indicates that physical activity can positively influence mental health and wellbeing (Biddle and Asare, 2011; Penedo and Dahn, 2005).

One area of mental health that has gained increasing interest in literature is mental toughness. Mental toughness represents a complex construct that encompasses an individual’s ability to handle stress and challenges, maintain focus, and persevere in the face of adversity (Clough et al., 2002). Mental toughness is increasingly recognized as a key factor in achieving personal and athletic goals. Recent research has explored the correlation between physical activity and mental toughness, with studies involving both males and females indicating that individuals who consistently participate in physical activities, such as trail running, tend to develop higher levels of mental toughness (Gameiro et al., 2023). Individuals possess greater mental toughness and demonstrate higher resilience, better emotional regulation, and enhanced performance in various domains, including sports, academics and the workplace (Gerber et al., 2013; Gucciardi et al., 2015). Gender affects mental toughness, with males exhibiting greater mental toughness than female participants (Mohebi et al., 2018), particularly when it comes to controlling emotions, life management, and self-confidence (Nicholls et al., 2009). Male participants have greater power to overcome challenges in a competition than female participants (Kumar and Ahmed, 2013).

In addition to its potential effect on mental toughness, participation in physical activities has also been associated with an improved quality of life, encompassing a comprehensive view of an individual’s wellbeing, including physical, mental, social, and environmental aspects (Pucci et al., 2012; Rejeski and Mihalko, 2001). Participating in consistent physical activities has been linked to higher levels of life satisfaction, better emotional and cognitive functioning, and enhanced social interactions (Bize et al., 2007). Early research suggests that gender-specific factors may influence the relationship between physical activity and various health outcomes (Eime et al., 2013). Rejeski and Mihalko demonstrated that individuals who engage in physical activity evaluate certain domains of quality of life as higher than those who are physically inactive (Rejeski and Mihalko, 2001). It seems that males might show a stronger inclination to engage in physical activities focused on improving both physical performance and mental toughness, while females may be more driven by the overall health and wellbeing benefits (Omorou et al., 2013). Lee et al. (2020) investigated gender-specific differences in quality of life. They concluded that males reported a higher quality of life than females across China, Ghana, India, Russia, and South Africa.

Prior research has explored the advantages of physical activity on mental toughness and quality of life. However, the potential differences between male and female participants have not been extensively examined. Gender-specific differences and associations among physical activity, mental toughness, and quality of life can significantly influence the design and implementation of targeted interventions. This cross-sectional study aimed to determine the effects of physical activity on mental health and quality of life in both male and female participants.

## Methods

### Study design

This research employed a cross-sectional study design.

### Ethical consideration

All participants submitted a written informed consent form, and the study received approval from the institutional review board at Imam Abdulrahman Bin Faisal University in Dammam.

### Participants

For this comparative study, 220 participants (110 males, 110 females) aged 18–31 were recruited from Imam Abdulrahman Bin Faisal University.

### Measures

#### Physical activity

The assessment of the participants’ physical activity levels was conducted utilizing the International Physical Activity Questionnaire-Short Form (IPAQ-SF). The participants reported the days and times of physical activity during the last 7 days. The IPAQ evaluates the frequency and duration of physical activity across different domains. This study measured MET-min for each participant and classified them (high, moderate, and low) based on their total weekly MET-min according to the IPAQ-SF analysis guidelines (Booth, 2000; Craig et al., 2003). Participants additionally reported the number of hours spent on sedentary behavior each day.

#### Mental toughness

Mental toughness was assessed using a questionnaire developed by Goldberg A.S. in 1998. This questionnaire consists of four subscales: handling pressure, concentration, mental rebounding, and winning attitude. It contains 80 statements, each offering two potential responses: True or False (Goldberg, 1998).

#### Quality of life

The assessment of participants’ quality of life was conducted using the World Health Organization Quality of Life-BREF (WHOQOL-BREF). The WHOQOL-BREF measures four domains of quality of life: physical health, mental health, social relationships, and environment (WHO, 2020).

### Procedure

The data collection occurred in a classroom setting. Before data collection, all relevant information on the research and how to provide their best-fit responses on the physical activity, mental toughness, and quality of life questionnaires was conveyed to the participants. Participants took 15–20 min to complete all the questionnaires. The anthropometric or sociodemographic data, along with their responses on outcome measures, were transferred to Excel sheets from the questionnaires. All the data were imported into SPSS software for further analysis.

### Statistical analysis

All the statistical analyses were carried out using the software known as IBM-Statistical Package for Social Sciences (IBM-SPSS), version 26.0, for Windows, Chicago, IL, United States. The data was analysed to identify any missing values or outliers. The normality assumption for the outcome measures was validated at a significance level of *p* < 0.05 using the Shapiro–Wilk test. Therefore, parametric tests were employed for further investigation. Gender-specific differences were assessed using an independent two-tailed *t*-test for physical activity, mental toughness, and quality of life. Cohen’s d was considered as the effect-size measure. The interpretation of effect size used as small (*d* = 0.2), medium (*d* = 0.5), and large (*d* = 0.8) based on benchmarks suggested by Cohen (1988). A one-way analysis of variance (ANOVA) was conducted to examine the differences in physical activity levels related to mental toughness and quality of life among male and female participants separately. Eta-squared served as the effect-size measure. The values of 0.01, 0.06, and 0.14 have been considered as benchmarks for small, medium, and large effects, respectively (Cohen, 1988). Due to multiple testing, all *post-hoc* comparisons were made with Bonferroni. The statistical significance level was set at 0.05.

## Results

Table 1 shows that there are significant differences between male and female participants for physical activity levels as high physical active participants (*t* = 4.21, *p* ≤ 0.001), moderate physical active participants (*t* = 3.72, *p* ≤ 0.001), and low physical active participants (*t* = 3.21, *p* = 0.002). Total scores of physically active participants (*t* = 7.38, *p* ≤ 0.001), and sitting time (*t* = 6.06, *p* ≤ 0.001). All the physical activity levels and the sitting time show a large effect size for both genders.

**Table 1:** Comparison between male and female participants for physical activity as per its levels.

PA level	Gender	*N*	Mean ± SD	t	Sig.	d
High	Male	57	5696.98 ± 861.37	4.21	<0.001	871.80
	Female	28	4850.50 ± 893.06			
Moderate	Male	37	3322.08 ± 1355.84	3.72	<0.001	1309.59
	Female	46	2246.89 ± 1271.37			
Low	Male	16	2398.3750 ± 535.08	3.21	0.002	520.91
	Female	36	1896.3056 ± 514.72			
Total scores	Male	110	4418.35 ± 1699.88	7.38	<0.001	1630.71
	Female	110	2794.89 ± 1558.47			
Sitting times	Male	110	440.00 ± 119.44	6.06	<0.001	117.33
	Female	110	344.21 ± 115.17			

Table 2 shows that there are statistically significant differences between gender for mental toughness parameters [handling pressure (*t* = 17.96, *p* ≤ 0.001), concentration (*t* = 16.07, *p* ≤ 0.001), mental rebounding (*t* = 13.34, *p* ≤ 0.001), winning attitude (*t* = 12.01, *p* ≤ 0.001)] and quality of life parameters [physical (*t* = 13.16, *p* ≤ 0.001), mental (*t* = 6.17, *p* ≤ 0.001), social (*t* = 4.58, *p* ≤ 0.001), environmental (*t* = 8.35, *p* ≤ 0.001)]. All the parameters for mental toughness and quality of life show a large effect size for both genders.

**Table 2:** Comparison between male and female participants for mental toughness and quality of life’s parameters.

Variables		Gender	*N*	Mean ± SD	*t*	Sig.	d
Mental toughness	Handling pressure	Male	110	16.53 ± 1.16	17.96	<0.001	1.25
		Female	110	13.49 ± 1.35			
	Concentration	Male	110	13.78 ± 1.16	16.07	<0.001	1.22
		Female	110	11.13 ± 1.29			
	Mental rebounding	Male	110	11.04 ± 1.39	13.34	<0.001	1.45
		Female	110	8.43 ± 1.51			
	Winning attitude	Male	110	6.25 ± 1.18	12.01	<0.001	1.17
		Female	110	4.34 ± 1.18			
Quality of life	Physical	Male	110	23.01 ± 1.34	13.16	<0.001	1.58
		Female	110	20.19 ± 1.80			
	Mental	Male	110	19.62 ± 1.59	6.17	<0.001	1.69
		Female	110	18.22 ± 1.77			
	Social	Male	110	16.78 ± 2.19	4.58	<0.001	2.05
		Female	110	15.52 ± 1.89			
	Environmental	Male	110	21.88 ± 5.41	8.35	<0.001	4.95
		Female	110	16.31 ± 4.44			

Table 3 showed that there are statistically significant differences between physical activity levels for mental toughness [handling pressure (*F* = 29.19, *p* ≤ 0.001), concentration (*F* = 54.39, *p* ≤ 0.001), mental rebounding (*F* = 63.72, *p* ≤ 0.001), winning attitude (*F* = 26.61, *p* ≤ 0.001)] and quality of life [physical (*F* = 7.83, *p* ≤ 0.001), mental (*F* = 12.94, *p* ≤ 0.001), social (*F* = 101.87, *p* ≤ 0.001), environmental (*F* = 418.58, *p* ≤ 0.001)] in male participants. All the parameters for mental toughness and quality of life show large effect sizes between physical activity levels for male participants. Only the physical aspect of quality of life shows a moderate effect size.

**Table 3:** Mental toughness and quality of life differences among male participants as per physical activity level classification.

Variables		PA level	*N*	Mean SD	F	Sig.	Eta-squared
Mental toughness	Handling pressure	High	57	17.11 ± 0.90	29.19	<0.001	0.353
		Moderate	37	16.22 ± 0.98			
		Low	16	15.19 ± 0.98			
	Concentration	High	57	14.53 ± 0.80	54.39	<0.001	0.504
		Moderate	37	13.24 ± 0.83			
		Low	16	12.38 ± 0.89			
	Mental rebounding	High	57	11.91 ± 0.95	63.73	<0.001	0.544
		Moderate	37	10.54 ± 1.02			
		Low	16	9.06 ± 0.77			
	Winning attitude	High	57	6.84 ± 0.94	29.61	<0.001	0.356
		Moderate	37	5.92 ± 1.01			
		Low	16	4.88 ± 0.89			
Quality of life	Physical	High	57	23.39 ± 1.42	7.83	<0.001	0.128
		Moderate	37	22.86 ± 1.13			
		Low	16	22.00 ± 0.89			
	Mental	High	57	20.26 ± 1.53	12.94	<0.001	0.195
		Moderate	37	19.11 ± 1.26			
		Low	16	18.50 ± 1.46			
	Social	High	57	16.00 ± 1.28	101.87	<0.001	0.656
		Moderate	37	19.11 ± 1.26			
		Low	16	14.19 ± 1.42			
	Environmental	High	57	25.84 ± 1.99	418.58	<0.001	0.887
		Moderate	37	14.81 ± 1.31			
		Low	16	24.13 ± 2.28			

Table 4 shows multiple comparisons of mental health and quality of life among male participants as per physical activity level classification. There are significant differences between physical activity levels for the parameters of mental toughness and quality of life, except between hyper and moderate physical activity level classification for quality of life’s physical parameter for male participants.

**Table 4:** Multiple comparisons of mental toughness and quality of life differences among male participants as per physical activity level classification.

Dependent variable			Mean difference (I-J)	Std. error	Sig.	95% CI	
						Lower bound	Upper bound
Handling pressure	Hyper	Moderate	0.89^*^	0.20	0.000	0.41	1.37
		Low	1.92^*^	0.27	0.000	1.27	2.56
	Moderate	Hyper	−0.89^*^	0.20	0.000	−1.37	−0.41
		Low	1.03^*^	0.28	0.001	0.35	1.71
	Low	Hyper	−1.92^*^	0.27	0.000	−2.56	−1.27
		Moderate	−1.03^*^	0.28	0.001	−1.71	−0.35
Concentration	Hyper	Moderate	1.28^*^	0.17	0.000	0.86	1.71
		Low	2.15^*^	0.23	0.000	1.58	2.72
	Moderate	Hyper	−1.28^*^	0.17	0.000	−1.71	−0.86
		Low	0.87^*^	0.25	0.002	0.27	1.47
	Low	Hyper	−2.15^*^	0.23	0.000	−2.72	−1.58
		Moderate	−0.87^*^	0.25	0.002	−1.47	−0.27
Mental rebounding	Hyper	Moderate	1.37^*^	0.20	0.000	0.88	1.86
		Low	2.85^*^	0.27	0.000	2.20	3.50
	Moderate	Hyper	−1.37^*^	0.20	0.000	−1.86	−0.88
		Low	1.48^*^	0.28	0.000	0.79	2.17
	Low	Hyper	−2.85^*^	0.27	0.000	−3.50	−2.20
		Moderate	−1.48^*^	0.28	0.000	−2.17	−0.79
Winning attitude	Hyper	Moderate	0.92^*^	0.20	0.000	0.43	1.41
		Low	1.97^*^	0.27	0.000	1.31	2.63
	Moderate	Hyper	−0.92^*^	0.20	0.000	−1.41	−0.43
		Low	1.04^*^	0.29	0.001	0.35	1.74
	Low	Hyper	−1.97^*^	0.27	0.000	−2.63	−1.31
		Moderate	−1.04^*^	0.29	0.001	−1.74	−0.35
Physical	Hyper	Moderate	0.52	0.27	0.162	−0.13	1.17
		Low	1.38^*^	0.36	0.001	0.51	2.26
	Moderate	Hyper	−0.52	0.27	0.162	−1.17	0.13
		Low	0.86	0.38	0.074	−0.06	1.79
	Low	Hyper	−1.38^*^	0.36	0.001	−2.26	−0.51
		Moderate	−0.86	0.38	0.074	−1.79	0.06
Mental	Hyper	Moderate	1.16^*^	0.30	0.001	0.42	1.89
		Low	1.76^*^	0.41	0.000	0.78	2.75
	Moderate	Hyper	−1.15^*^	0.30	0.001	−1.89	−0.42
		Low	0.61	0.43	0.480	−0.44	1.65
	Low	Hyper	−1.76^*^	0.41	0.000	−2.75	−0.78
		Moderate	−0.61	0.43	0.480	−1.65	0.44
Social	Hyper	Moderate	−3.11^*^	0.27	0.000	−3.77	−2.44
		Low	1.81^*^	0.37	0.000	0.92	2.71
	Moderate	Hyper	3.11^*^	0.27	0.000	2.44	3.77
		Low	4.92^*^	0.39	0.000	3.98	5.86
	Low	Hyper	−1.81^*^	0.37	0.000	−2.71	−0.92
		Moderate	−4.92^*^	0.39	0.000	−5.86	−3.98
Environmental	Hyper	Moderate	11.03^*^	0.39	0.000	10.09	11.97
		Low	1.72^*^	0.52	0.004	0.45	2.98
	Moderate	Hyper	−11.03^*^	0.39	0.000	−11.97	−10.09
		Low	−9.31^*^	0.55	0.000	−10.65	−7.98
	Low	Hyper	−1.71^*^	0.52	0.004	−2.98	−0.45
		Moderate	9.31^*^	0.55	0.000	7.98	10.65

*The mean difference is significant at the 0.05 level.

Table 5 shows that there are statistically significant differences between physical activity levels for mental toughness parameters [handling pressure (*F* = 40.95, *p* ≤ 0.001), concentration (*F* = 44.63, *p* ≤ 0.001), mental rebounding (*F* = 44.12, *p* ≤ 0.001), winning attitude (*F* = 17.45, *p* ≤ 0.001)] and quality of life parameters [physical (*F* = 17.23, *p* ≤ 0.001), mental (*F* = 24.58, *p* ≤ 0.001), social (*F* = 40.39, *p* ≤ 0.001), environmental (*F* = 36.69, *p* ≤ 0.001)] in female participants. All the parameters for mental toughness and quality of life show large effect sizes between physical activity levels for female participants.

**Table 5:** Mental toughness and quality of life differences among female participants as per physical activity level classification.

Variables		PA level	*N*	Mean SD	F	Sig.	Eta-Squared
Mental toughness	Handling pressure	High	28	14.89 ± 0.96	40.95	<0.001	0.434
		Moderate	46	13.35 ± 0.99			
		Low	36	12.58 ± 1.11			
	Concentration	High	28	12.54 ± 0.58	44.63	<0.001	0.455
		Moderate	46	10.91 ± 1.11			
		Low	36	10.31 ± 0.98			
	Mental rebounding	High	28	9.96 ± 0.92	44.12	<0.001	0.452
		Moderate	46	8.37 ± 1.24			
		Low	36	7.31 ± 1.12			
	Winning attitude	High	28	4.82 ± 0.86	19.45	<0.001	0.267
		Moderate	46	4.72 ± 1.05			
		Low	36	3.47 ± 1.08			
Quality of life	Physical	High	28	21.68 ± 1.52	17.23	<0.001	0.244
		Moderate	46	19.85 ± 1.58			
		Low	36	19.47 ± 1.63			
	Mental	High	28	19.82 ± 1.25	24.58	<0.001	0.315
		Moderate	46	18.00 ± 1.53			
		Low	36	17.25 ± 1.57			
	Social	High	28	15.07 ± 1.41	40.39	<0.001	0.430
		Moderate	46	14.43 ± 1.34			
		Low	36	17.25 ± 1.57			
	Environmental	High	28	23.32 ± 2.25	36.69	<0.001	0.871
		Moderate	46	14.43 ± 1.34			
		Low	36	13.25 ± 1.32			

Table 6 presents multiple comparisons of mental health and quality of life among female participants based on physical activity level classification. Significant differences exist between physical activity levels concerning mental toughness and quality of life parameters, except for the comparison between hyper and moderate physical activity classifications regarding winning attitude and between moderate and low physical activity classifications regarding the physical and mental aspects of quality of life in female participants.

**Table 6:** Multiple comparisons of mental toughness and quality of life differences among female participants as per physical activity level classification.

Bonferroni							
Dependent variable	(I)	(J)	Mean difference (I-J)	Std. error	Sig.	95% CI	
						Lower bound	Upper bound
Handling pressure	Hyper	Moderate	1.55^*^	0.25	<0.001	0.95	2.14
		Low	2.31^*^	0.26	<0.001	1.68	2.94
	Moderate	Hyper	−1.55^*^	0.25	<0.001	−2.14	−0.95
		Low	0.76^*^	0.23	0.003	0.21	1.32
	Low	Hyper	−2.31^*^	0.26	<0.001	−2.94	−1.68
		Moderate	−0.76^*^	0.23	0.003	−1.32	−0.21
Concentration	Hyper	Moderate	1.62^*^	0.23	<0.001	1.06	2.18
		Low	2.23^*^	0.24	<0.001	1.64	2.82
	Moderate	Hyper	−1.62^*^	0.23	<0.001	−2.18	−1.06
		Low	0.61^*^	0.21	0.016	0.09	1.13
	Low	Hyper	−2.23^*^	0.24	<0.001	−2.82	−1.64
		Moderate	−0.61^*^	0.21	0.016	−1.13	−0.09
Mental rebounding	Hyper	Moderate	1.59^*^	0.27	<0.001	0.94	2.25
		Low	2.66^*^	0.28	<0.001	1.97	3.35
	Moderate	Hyper	−1.59^*^	0.27	<0.001	−2.25	−0.94
		Low	1.06^*^	0.25	<0.001	0.46	1.67
	Low	Hyper	−2.66^*^	0.28	<0.001	−3.35	−1.97
		Moderate	−1.06^*^	0.25	<0.001	−1.67	−0.46
Winning attitude	Hyper	Moderate	0.10	0.24	1	−0.49	0.70
		Low	1.35^*^	0.26	<0.001	0.73	1.97
	Moderate	Hyper	−0.10	0.24	1	−0.70	0.49
		Low	1.25^*^	0.23	<0.001	0.70	1.79
	Low	Hyper	−1.35^*^	0.26	<0.001	−1.97	−0.73
		Moderate	−1.25^*^	0.23	<0.001	−1.79	−0.70
Physical	Hyper	Moderate	1.83^*^	0.38	<0.001	0.91	2.75
		Low	2.21^*^	0.40	<0.001	1.24	3.17
	Moderate	Hyper	−1.83^*^	0.38	<0.001	−2.75	−0.91
		Low	0.37	0.35	0.863	−0.48	1.23
	Low	Hyper	−2.21^*^	0.40	<0.001	−3.17	−1.24
		Moderate	−0.37	0.35	0.863	−1.23	0.48
Mental	Hyper	Moderate	1.82^*^	0.36	<0.001	0.96	2.69
		Low	2.57^*^	0.37	<0.001	1.66	3.48
	Moderate	Hyper	−1.82^*^	0.36	<0.001	−2.69	−0.96
		Low	0.75	0.33	0.075	−0.05	1.55
	Low	Hyper	−2.57^*^	0.37	<0.001	−3.48	−1.66
		Moderate	−0.75	0.33	0.075	−1.55	0.05
Social	Hyper	Moderate	0.64	0.35	0.204	−0.20	1.48
		Low	−2.18^*^	0.36	<0.001	−3.06	−1.30
	Moderate	Hyper	−0.64	0.35	0.204	−1.48	0.20
		Low	−2.82^*^	0.32	<0.001	−3.59	−2.04
	Low	Hyper	2.18^*^	0.36	<0.001	1.30	3.06
		Moderate	2.82^*^	0.32	<0.001	2.04	3.59
Environmental	Hyper	Moderate	8.89^*^	0.39	<0.001	7.95	9.83
		Low	10.07^*^	0.41	<0.001	9.08	11.06
	Moderate	Hyper	−8.89^*^	0.39	<0.001	−9.83	−7.95
		Low	1.18^*^	0.36	0.004	0.31	2.06
	Low	Hyper	−10.07^*^	0.41	<0.001	−11.06	−9.08
		Moderate	−1.18^*^	0.36	0.004	−2.06	−0.31

*The mean difference is significant at the 0.05 level.

## Discussion

This research aimed to investigate the effect of physical activity on mental toughness and quality of life among male and female participants. To achieve the research objective, data were collected using distinct questionnaires, including the IPAQ, Mental Toughness Scale, and WHOQOL-BREF. The findings revealed significant differences in physical activity levels among male and female participants: high (*t* = 4.21, *p* ≤ 0.001), moderate (*t* = 3.72, *p* ≤ 0.001), and low (*t* = 3.21, *p* = 0.002). Moreover, statistically significant differences between genders also existed for mental toughness parameters: handling pressure (*t* = 17.96, *p* ≤ 0.001), concentration (*t* = 16.07, *p* ≤ 0.001), mental rebounding (*t* = 13.34, *p* ≤ 0.001), and winning attitude (*t* = 12.01, *p* ≤ 0.001), as well as for quality of life parameters: physical (*t* = 13.16, *p* ≤ 0.001), mental (*t* = 6.17, *p* ≤ 0.001), social (*t* = 4.58, *p* ≤ 0.001), and environmental (*t* = 8.35, *p* ≤ 0.001). These findings are consistent with those of other researchers, as mentioned below.

Bergier et al. (2015) compared the levels of physical activity in males and females. They confirmed a more positive assessment of physical activity, as a high level of activity was recorded in males (57.1%) and in females (45.7%). In contrast, low activity was reported in males (3.6%) and females (6.3%). An assessment of physical activity levels among Ukrainian university students is promising because over 90% of the respondents reported high or moderate activity, while only a small percentage reported low activity (Bergier et al., 2015). A high level of physical activity was noted among males (20.9%) and females (17.3%), whereas a low level of physical activity was recorded among females (36.3%) and males (20.9%) (Peltzer and Pengpid, 2013). In the present study, high activity was reported in males (51.81%) and in females (25.45%), while low activity was reported in males (14.55%) and in females (32.73%). A total of 76.36% of respondents reported high or moderate physical activity, while 23.64% reported low activity.

The examination of physical activity differences between male and female students reveals a multifaceted landscape influenced by psychosocial factors, social support, and the nature of the physical activities undertaken. Kljajević et al. (2022) conducted a systematic review assessing physical fitness levels and physical activity among university students, indicating that males generally participate in more intense physical activity than females (Kljajević et al., 2022). These differences are further elaborated by Valis and Gonzalez (2017), who found that male university students reported higher levels of vigorous occupational physical activity and overall physical activity, suggesting inherent gender differences in activity preferences and engagement. Kretschmer et al. (2023) investigated the evidence across nine countries for physical activity differences by gender. They reported that higher mean volumes of moderate to vigorous physical activity were found in male participants, along with greater variability than in female participants. The results indicated minimal to no difference in participation between males and females regarding the distribution of light-intensity or sedentary activities. Research has shown that differences in physical activity levels exist across various gender groups, encompassing children, adolescents, adults, and older individuals, with findings consistently indicating that males tend to participate in higher levels of physical activity than their female counterparts (Azevedo et al., 2007; Trost et al., 2002).

Mental toughness is a multidimensional construct often highlighted as an essential component of successful performance (Ahsan and Mohammad, 2017; Golby and Sheard, 2004). Nicholls et al. (2008) revealed a significant difference in mental toughness between male and female participants. It was reported that male subjects exhibited a higher level of mental toughness than their female counterparts. Fisher et al. (2021) identify that men often use problem-based coping strategies and demonstrate self-reliance, which are elements of mental toughness, potentially reducing the likelihood of anxiety and depression among males. Newland et al. (2013) indicated a significant relationship between the mental toughness of male subjects and their performance, while this relationship was not observed in female subjects. Nicholls et al. (2008) investigated the mental toughness among athletes and found that males scored significantly higher than females on total mental toughness with low effect size (Cohen’s *d* = 0.33). Putra et al. (2024) investigate the differences in mental toughness between genders and conclude that males have higher average mental toughness scores than females. They also elaborate that male participants generally exhibit better mental toughness, making them less susceptible to experiencing excessive psychological disorders such as stress and depression. Our findings align with existing literature and demonstrate that mental toughness is a more robust predictor of approach quality of life and physical activity. Participation in physical activities may also enhance mental toughness and quality of life in females.

The literature reveals that gender differences significantly affect quality of life across various health conditions. Females generally report a lower quality of life in areas related to mental health, physical health, and chronic disease management compared to males. The impact of physical activity on quality of life also exhibits gender-specific patterns. Nowak et al. (2019) observed that certain types of physical activity positively correlate with the quality of life among university students, with notable differences in activity patterns and quality of life outcomes between males and females. Additionally, Dos Santos et al. (2021) demonstrated that creatine supplementation, in conjunction with resistance training, significantly improves muscle strength in older females, potentially enhancing their physical quality of life. Mental health is another critical area where gender differences influence quality of life. Females are at a higher risk of experiencing depressive symptoms compared to their male counterparts, highlighting the need for gender-specific mental health interventions in the workplace (Torquati et al., 2019). Additionally, Mental health professionals, especially women, are more vulnerable to secondary traumatization, which can significantly impact their quality of life and professional effectiveness (Baum, 2016). Van Den Eijnden et al. (2018) discovered that engagement with games and social media adversely impacts psychosocial wellbeing and academic performance, with gender differences shaping these results. Fergus et al. (2024) further elaborated on psychological wellbeing, emphasizing gender-based variations in wellness dimensions that enhance overall quality of life. The evidence underscores the need for gender-specific healthcare strategies that address not only the biological differences but also the socio-cultural and psychological dimensions that differentially affect males and females.

This study has some limitations. It utilized a cross-sectional approach, which restricts the ability to establish causality between physical activity, mental toughness, and quality of life. Longitudinal studies would provide more robust evidence of these relationships. A small sample size may reduce statistical power and impact the reliability of gender comparisons. Additionally, reliance on self-reported measures for physical activity, mental toughness, and quality of life could introduce response biases, and objective measures (e.g., accelerometers for physical activity) could enhance validity. The findings may not be generalizable to wider populations, as the data were gathered from only one institution. Factors such as diet, sleep, stress levels, and socioeconomic status were not controlled, which could influence mental toughness and quality of life independently of physical activity. The study might not account for cultural differences in perceptions of mental toughness or quality of life, which could vary across regions. Furthermore, the use of the questionnaires and scales may not capture all dimensions of mental toughness or quality of life, leading to incomplete assessments.

## Conclusion

Statistically significant differences were found between genders in levels of physical activity, mental toughness, and quality of life. Future research should aim to unravel the complex interlink between physical activity, mental toughness, and quality of life, using longitudinal designs and diverse populations to validate the findings and explore the underlying mechanisms further. Such efforts will advance the development of holistic, gender-responsive interventions that promote mental toughness and quality of life for both males and females.

## Data Availability

The raw data supporting the conclusions of this article will be made available by the authors, without undue reservation.
